# Laryngeal sensitivity evaluation and dysphagia: Hospital Sírio-Libanês experience

**DOI:** 10.1590/S1516-31802004000500004

**Published:** 2004-09-01

**Authors:** Orlando Parise, Roberto Elias Villela Miguel, Daniela Luci D'Aquino Gomes, Antonio Douglas Menon, Kiyoshi Hashiba

**Keywords:** Larynx, Dysphagia, Deglutation disorders, Gastroesophageal reflux, Language and hearing sciences speech, Laringe, Disfagia, Transtorno de deglutição, Refluxo gastroesofágico, Fonoaudiologia

## Abstract

**CONTEXT:**

Laryngeal sensitivity is important in the coordination of swallowing coordination and avoidance of aspiration.

**OBJECTIVE:**

To briefly review the physiology of swallowing and report on our experience with laryngeal sensitivity evaluation among patients presenting dysphagia.

**TYPE OF STUDY:**

Prospective.

**SETTING:**

Endoscopy Department, Hospital SírioLibanês.

**METHODS:**

Clinical data, endoscopic findings from the larynx and the laryngeal sensitivity, as assessed via the Flexible Endoscopic Evaluation of Swallowing with Sensory Testing (FEESST) protocol (using the Pentax AP4000 system), were prospectively studied. The chi-squared and Student t tests were used to compare differences, which were considered significant if p < or = 0.05.

**RESULTS:**

The study included 111 patients. A direct association was observed for hyperplasia and hyperemia of the posterior commissure region in relation to globus (p = 0.01) and regurgitation (p = 0.04). Hyperemia of the posterior commissure region had a direct association with sialorrhea (p = 0.03) and an inverse association with xerostomia (p = 0.03). There was a direct association between severe laryngeal sensitivity deficit and previous radiotherapy of the head and neck (p = 0.001).

**DISCUSSION:**

These data emphasize the association between proximal gastroesophageal reflux and chronic posterior laryngitis, and suggest that decreased laryngeal sensitivity could be a side effect of radiotherapy.

**CONCLUSIONS:**

Even considering that these results are preliminary, the endoscopic findings from laryngoscopy seem to be important in the diagnosis of proximal gastroesophageal reflux. Study of laryngeal sensitivity may have the potential for improving the knowledge and clinical management of dysphagia.

## RESUMO

**CONTEXTO:**

A sensibilidade da laringe é importante para coordenar a deglutição e prevenir a aspiração.

**OBJETIVO:**

Revisar os mecanismos fisiológicos de coordenação da deglutição e reportar a experiência do Hospital Sírio Libanês com a avaliação da sensibilidade da laringe nos pacientes com disfagia.

**TIPO DE ESTUDO:**

Prospectivo.

**LOCAL:**

Instituição privada (Departamento de Endoscopia do Hospital Sírio Libanês, São Paulo, Brasil), pacientes ambulatoriais.

**MATERIAL E MÉTODOS:**

Os dados clínicos, os achados endoscópicos na laringe e a sensibilidade da laringe avaliada pelo protocolo FEESST (Flexible Endoscopic Evaluation of Swallowing with Sensory Testing) utilizando o sistema Pentax AP4000 foram analizados prospectivamente. Diferenças foram comparadas pelo teste de t de Student ou pelo teste do chi-quadrado, sendo consideradas significantes diferenças com o p < ou = 0,05.

**RESULTADOS:**

111 pacientes foram incluídos no estudo. Houve uma associação direta entre hiperplasia e hiperemia da região da comissura posterior com historia de globus histérico (p = 0,01) e regurgitação (p = 0,04); associação direta entre hiperemia da região da comissura posterior com sialorréia (p = 0,03) e associação inversa com xerostomia (p = 0,03); e associação direta entre déficit grave da sensibilidade da laringe e radioterapia prévia na cabeça e pescoço (p = 0,001).

**DISCUSSÃO:**

Estes dados enfatizam a associação entre refluxo gastroesofágico proximal e a laringite posterior crônica; e sugerem que a radioterapia tem como um efeito colateral a redução da sensibilidade da laringe.

**CONCLUSÕES:**

Mesmo considerando que estes resultados são preliminares, o aspecto laringoscópico da laringe é importante para o diagnóstico do refluxo gastroesofágico proximal e o estudo da sensibilidade da laringe pode aumentar nosso conhecimento e melhorar o manejo clínico da disfagia.

## INTRODUCTION

Laryngeal sensitivity is important for survival. Such sensitivity enables food to be swallowed without aspiration, and if this should occur (or if food should penetrate the laryngeal vestibule), coughing will expel the food back to the oropharynx.

The act of swallowing can be divided into three parts: the first (oral) is voluntary and the next two (pharyngeal and esophageal) are reflexes based on laryngeal sensitivity. When food is pushed backwards by the tongue, towards the soft palate and oropharynx, the epiglottis directs the food toward the two sides of the oropharynx. It is essential at this stage that the vocal folds and cords close in a coordinated fashion and the pyriform sinuses open for the food to be correctly directed to the cervical esophagus. Sensitivity of the larynx is the trigger that sets off this reflex mechanism, thereby resulting in swallowing and protection of the airway.

This reflex of closing the glottis is basically set off by receptors located in the aryepiglottic fold, arytenoid and laryngeal vestibule.^1,2^ The concentration of such receptors in these regions is often greater than in other regions of the pharynx. The elderly demonstrate progressive loss of laryngeal sensitivity, which is probably a degenerative mechanism of aging.^3^ The aim of this study was to evaluate the findings from laryngoscopy and the Flexible Endoscopic Evaluation of Swallowing with Sensory Testing (FEESST)^4-6^ protocol among patients with dysphagia, in relation to clinical data.

## METHODS

*Type of study*: Prospective, non-comparative study.

*Setting*: Private institution, outpatients. *Sample*: The study included 111 patients with dysphagia and/or other proximal gastroesophageal reflux disease (GERD) symptoms, without systemic or topical laryngeal analgesia.

*Procedures*: The Flexible Endoscopic Evaluation of Swallowing with Sensory Test (FEESST) protocol was utilized to quantitatively evaluate laryngeal sensitivity. This protocol evaluates the sensitivity threshold for triggering the reflex of closing the glottis and swallowing, from a functional point of view (mechanical effectiveness). The test is based on the emission of an air stream through a nozzle in the flexible laryngoscope, at an adjustable pressure of 2 to 10 mmHg, in pulses lasting 50 milliseconds each or with continuous airflow. The test is performed without any local or systemic anesthesia, thereby avoiding any change in sensitivity of the airway mucosa. Individuals with sensitivity preserved react to the air pulse at pressures of less than 4 mmHg. If the reaction only occurs between 4 and 6 mmHg, this indicates a moderate sensitivity deficit and if it only occurs at pressures of more than 6 mmHg, the sensitivity deficit is severe. Functional evaluation of swallowing is accomplished by positioning the tip of the fiber of the same endoscope in the oropharynx and directly viewing the swallowing of foods of various consistencies that have been stained.

*Main measurements*: Clinical data, endoscopic findings from the larynx and laryngeal sensitivity assessed via the FEESST protocol.

*Statistical Methods*: The Fisher exact and chi-squared tests were used to compare differences in larynx sensitivity in relation to clinical and endoscopic data. Differences with p ≤ 0.05 were considered significant.

## RESULTS

The mean patient age was 59 years (range 17-89). Fifty-six patients said they were regular consumers of alcohol and 32 patients were smokers. In addition to dysphagia, 63 patients mentioned that they frequently needed to clear their throats, 57 had globus, 52 chronic cough, 48 retrosternal pyrosis, 44 regurgitation and 25 asthma.

With regard to the endoscopic signs of proximal gastroesophageal reflux disease, 86 patients presented hyperemia of the posterior larynx, 82 had hyperplasia of the interarytenoid region and 43 had both signs.

All 111 patients evaluated in this study underwent laryngoscopy and FEESST without any kind of anesthesia (topical, local or systemic), with no problems. Twenty-seven patients presented normal laryngeal sensitivity, 19 had a moderate deficit, 57 had a severe laryngeal sensitivity deficit and for eight patients the FEESST was not conclusive.

No significant association was found for age, alcohol consumption or smoking in relation to the laryngeal findings from laryngoscopy or FEESST. A direct association was observed for hyperplasia and hyperemia of the posterior commissure in relation to globus (p = 0.01; Table 1) and regurgitation (p = 0.04; Table 1); hyperemia of the posterior commis-sure had a direct association with sialorrhea (p = 0.03, Table 2) and an inverse association with xerostomia (p = 0.03, Table 2).

**Table 1:** Direct association of hyperplasia and hyperemia of the posterior commissure with globus (p = 0.01) and regurgitation (p = 0.04) in 111 patients with symptoms of dysphagia and/or gastroesophageal reflux*

	With globus	Without globus	With regurgitation	Without regurgitation
Without hyperemia and hyperplasia	19	12	22	9
With hyperemia and hyperplasia	24	45	34	35

* *data are missing for 11 patients.*

**Table 2:** Direct association between hyperemia of the posterior commissure with sialorrhea (p = 0.03) and inverse association with xerostomia (p = 0.03) in 111 patients with symptoms of dysphagia and/or gastroesophageal reflux*

	Without sialorrhea	With sialorrhea	Without xerostomia	With xerostomia
Without hyperemia	19	1	15	5
With hyperemia	56	30	82	2

* *data are missing for 5 and 7 patients.*

Twelve patients had been submitted to radiotherapy, with the larynx included within the field of treatment, during the 36 months preceding FEESST. Eleven of these patients presented no laryngeal sensitivity upon stimulus using a pressure of 10 mmHg (Table 3), thereby showing a direct association between severe laryngeal sensitivity deficit and prior radiotherapy of the head and neck (p = 0.001).

**Table 3:** Direct association between previous radiotherapy of the head and neck and severe laryngeal sensitivity deficit (p = 0.001) in 111 patients with symptoms of dysphagia and/or gastroesophageal reflux

	Normal sensitivity or moderate deficit	Severe deficit	Inconclusive FEESST	Total
Without radiotherapy	53	38	8	99
With radiotherapy	1	11	0	12
**Total**	**54**	**49**	**8**	**111**

*FEESST = Flexible Endoscopic Evaluation of Swallowing with Sensory Testing.*

## DISCUSSION

This study evaluated the association between the clinical data for patients complaining of dysphagia and the laryngoscopy findings and FEESST. In addition to their dysphagia, the high incidence of a frequent need to clear the throat, globus, chronic cough, retrosternal pyrosis, regurgitation and asthma suggested that there was significant prevalence of proximal gastroesophageal reflux in this sample. This hypothesis was supported by the laryngoscopic findings, in particular the association observed for hyperplasia and hyperemia of the posterior commissure in relation to globus and regurgitation.^7,8^ It has been suggested that laryngeal findings could form an alternative to 24-hour pH monitoring for diagnosing proximal gastroesophageal reflux.^9^ Direct observation of swallowing by means of a flexible fiberoptic could be enough for diagnosing dysphagia.^10^

The FEESST test used on patients with dysphagia has demonstrated correlations between severe laryngeal sensitivity deficit and aspiration,^11,12^ penetration and the presence of food residue after swallowing.^12^ Likewise, severe laryngopharyngeal sensitivity deficit has been shown among patients with dysphagia,^11^ even if compared with such deficit among healthy controls.^13^ In another prospective randomized study, with the objective of comparing FEESST with the swallowing of barium contrast, for the control of dysphagia patients, no difference in the incidence of aspiration pneumonia between the two groups was observed.^14^ On the other hand, in a study among post-stroke neurological patients presenting dysphagia,^15^ there was a marked laryngeal sensitivity deficit on the affected side, in comparison with these patients’ opposite side and also with the sensitivity shown by controls of the same age.^16^ These same neurological patients also revealed that reduced laryngeal sensitivity is often not only asymptomatic but also predisposes towards aspiration and pneumonia.^17^ Our study did not established an association between laryngeal sensitivity deficit and asthma.

Among the potential benefits and advantages of laryngeal sensitivity analysis via FEESST, in comparison with barium swallow studies (the present standard for the evaluation of swallowing), are the facts that FEESST is done at the bedside, in the patient's feeding position; air is used instead of barium, without exposure to any radiation; and both the sensitivity and the motor component of the pharynx are analyzed at a much lower cost. The disadvantage is the lack of information regarding the esophageal motor component, which is visible only via a barium swallow study.

Probably the most important finding from the present study was the direct association that was observed between severe laryngeal sensitivity deficit and prior radiotherapy of the head and neck. At present, there is a tendency to treat advanced laryngeal carcinoma using protocols aiming at organ preservation. Even in cases in which partial surgery would be suitable, a combination of chemotherapy and radiotherapy is often chosen. The present data give an additional argument in the discussion regarding the indication of organ preservation, considering that it is not clear what kind of long-term consequences the lack of laryngeal sensitivity would bring to patients whose larynxes are preserved without sensitivity. Protocols that preserve the larynx are a sound option in the treatment of laryngeal carcinoma, but we do not today have any means of evaluating the impact of losing laryngeal sensitivity due to organ preservation treatment among patients in a poor respiratory condition.

## CONCLUSIONS

Even considering that these results are preliminary, the data emphasize the association between the symptoms of proximal gastroesophageal reflux (globus, regurgitation and sialorrhea, with the latter taken to be an attempt at buffering the proximal reflux) and the presence of chronic posterior laryngitis (hyperplasia and hyperemia of the posterior commissure).

Specifically, the information that radio-therapy significantly reduces laryngeal sensitivity leads us to ponder whether the adoption of aggressive protocols for organ preservation in the head and neck is risky for those with limited pulmonary function such as the elderly or emphysematous patients.

The study of laryngeal sensitivity remains a challenge. Its utilization in clinical practice may improve our knowledge of the physiology of swallowing and standardize the management of dysphagia.
